# scLTdb: a comprehensive single-cell lineage tracing database

**DOI:** 10.1093/nar/gkae913

**Published:** 2024-10-29

**Authors:** Junyao Jiang, Xing Ye, Yunhui Kong, Chenyu Guo, Mingyuan Zhang, Fang Cao, Yanxiao Zhang, Weike Pei

**Affiliations:** Westlake Laboratory of Life Sciences and Biomedicine, No. 18 Shilongshan Road, Hangzhou 310024, Zhejiang, China; School of Life Sciences, Westlake University, No. 600 Dunyu Road, Hangzhou 310030, Zhejiang, China; Westlake Institute for Advanced Study, No. 18 Shilongshan Road, Hangzhou 310024, Zhejiang, China; Westlake Laboratory of Life Sciences and Biomedicine, No. 18 Shilongshan Road, Hangzhou 310024, Zhejiang, China; School of Life Sciences, Westlake University, No. 600 Dunyu Road, Hangzhou 310030, Zhejiang, China; Division of Life Sciences and Medicine, University of Science and Technology of China, No. 96 Jinzhai Road, Hefei 230027, Anhui, China; Institute of Modern Biology, Nanjing University, No. 163 Xianlin Road, Nanjing 210008, Jiangsu, China; Westlake Laboratory of Life Sciences and Biomedicine, No. 18 Shilongshan Road, Hangzhou 310024, Zhejiang, China; School of Life Sciences, Westlake University, No. 600 Dunyu Road, Hangzhou 310030, Zhejiang, China; Westlake Institute for Advanced Study, No. 18 Shilongshan Road, Hangzhou 310024, Zhejiang, China; School of Life Sciences, Fudan University, No. 2005 Songhu Road, Shanghai 200438, China; Westlake Laboratory of Life Sciences and Biomedicine, No. 18 Shilongshan Road, Hangzhou 310024, Zhejiang, China; School of Life Sciences, Westlake University, No. 600 Dunyu Road, Hangzhou 310030, Zhejiang, China; Westlake Institute for Advanced Study, No. 18 Shilongshan Road, Hangzhou 310024, Zhejiang, China; Department of Neurosurgery, The First Affiliated Hospital of Hainan Medical University, No. 31 Longhua Road, Haikou 570100, Hainan, China; Westlake Laboratory of Life Sciences and Biomedicine, No. 18 Shilongshan Road, Hangzhou 310024, Zhejiang, China; School of Life Sciences, Westlake University, No. 600 Dunyu Road, Hangzhou 310030, Zhejiang, China; Westlake Institute for Advanced Study, No. 18 Shilongshan Road, Hangzhou 310024, Zhejiang, China; Research Center for Industries of the Future, Westlake University, No. 600 Dunyu Road, Hangzhou 310030, Zhejiang, China; Westlake Laboratory of Life Sciences and Biomedicine, No. 18 Shilongshan Road, Hangzhou 310024, Zhejiang, China; School of Life Sciences, Westlake University, No. 600 Dunyu Road, Hangzhou 310030, Zhejiang, China; Westlake Institute for Advanced Study, No. 18 Shilongshan Road, Hangzhou 310024, Zhejiang, China; Research Center for Industries of the Future, Westlake University, No. 600 Dunyu Road, Hangzhou 310030, Zhejiang, China

## Abstract

Single-cell lineage tracing (scLT) is a powerful technique that integrates cellular barcoding with single-cell sequencing technologies. This new approach enables the simultaneous measurement of cell fate and molecular profiles at single-cell resolution, uncovering the gene regulatory program of cell fate determination. However, a comprehensive scLT database is not yet available. Here, we present the single-cell lineage tracing database (scLTdb, https://scltdb.com) containing 109 datasets that are manually curated and analyzed through a standard pipeline. The scLTdb provides interactive analysis modules for visualizing and re-analyzing scLT datasets, especially the comprehensive cell fate analysis and lineage relationship analysis. Importantly, scLTdb also allows users to identify fate-related gene signatures. In conclusion, scLTdb provides an interactive interface of scLT data exploration and analysis, and will facilitate the understanding of cell fate decision and lineage commitment in development and diseases.

## Graphical Abstract

**Graphical Abstract:**
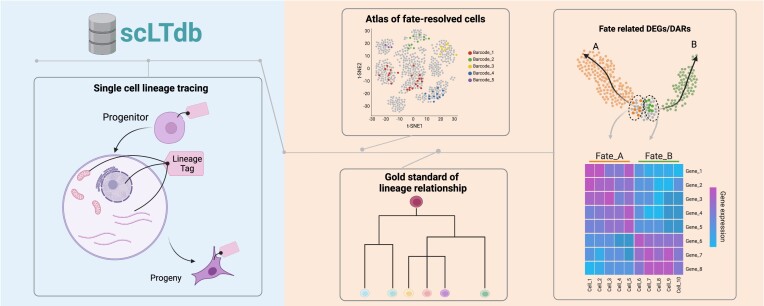

## Introduction

A fundamental aim in stem cell and developmental biology is to accurately resolve the developmental history and cell fate of individual cells. To this aim, lineage tracing is widely used to provide key information about the location, number and cell state of founder cells and their descendants by identifying all progeny of a single cell in tissues (1,2). Conventional lineage tracing has involved fluorescent proteins to genetically label progenitors and track the behavior of their progeny cells *in vivo* (1). It has provided new insights into the mechanisms of stem cell fate determination during tissue development, maintenance and regeneration (3,4). However, conventional lineage tracing approaches are limited by either invasive manipulation of cells (e.g. transplantation) that may perturb the physiology of cells, or by limited numbers of fluorescent reporters that cannot quantitatively analyze massive numbers of cells at single-cell resolution (5).

**Figure 1.**
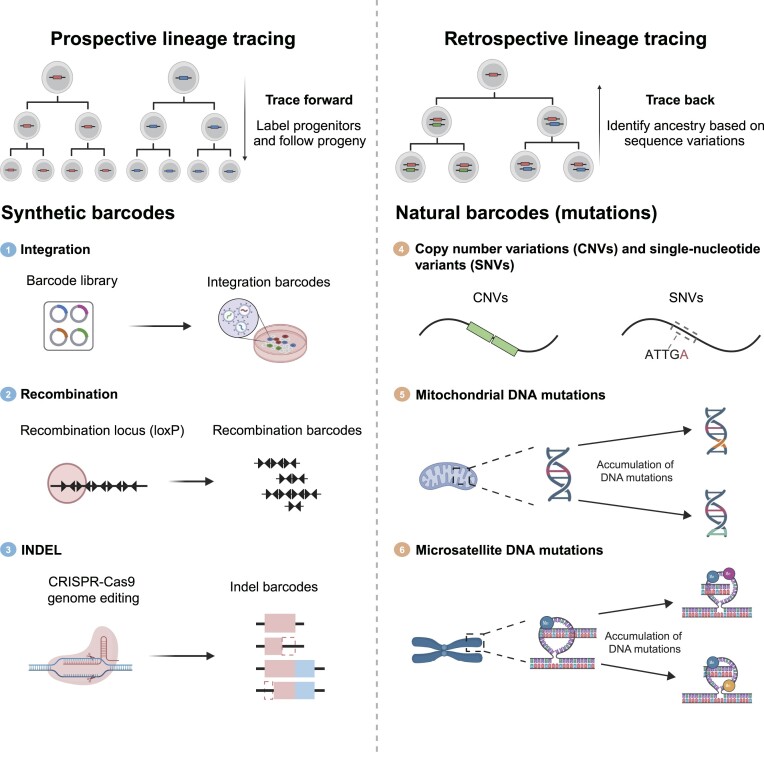
Introduction of scLT technologies and barcode types.

In recent years, the next-generation lineage tracing has involved cellular barcoding that uses a large number of synthetic DNA sequences to uniquely label cells (prospective lineage tracing), providing quantitative insights into the stem cell dynamics and cell fate outcomes (6,7). This strategy can be achieved using various barcode types, including integration of exogenous random DNA sequences into the cell genome, recombination of endogenous DNA units or genome editing-mediated DNA insertions and deletions (INDEL) (Figure 1) (5). Cellular barcoding allows researchers to distinguish individual cells based on specific DNA barcode sequences at clonal resolution (8). Combining cellular barcoding with single-cell genomics, also known as single-cell lineage tracing (scLT), has generated rich datasets that resolve cell fate and transcriptional or epigenetic state of the same cells in parallel (9–11). The emergence of scLT has begun to reveal the gene regulatory program of cell fate decision in various tissues, such as central nervous system and hematopoietic system, and to dissect the mechanism underlying clonal behavior of cancer cells during tumor formation, metastasis and drug resistance (12–16). As a result, scLT is providing insights to precisely manipulate cell fate *in vivo* and to predict the cellular origins of cancer (17).

Now, using mutations and DNA variations that accumulated in cells as natural barcodes, scLT is used to reconstruct high-resolution lineage trees in human hematopoiesis and cancers (retrospective lineage tracing), which were lacking in medical research (18–20). As an emerging technique, scLT is beginning to refine our understanding of disease pathology, particularly deciphering clonal contributions in human diseases. Given the significance of scLT in biology and medicine, the publicly available scLT data call for in-depth and integrated analysis to yield new insights on the cell fate determination in development and diseases.

Until now, there is no public scLT database. To fill this gap, we developed single-cell lineage tracing database (scLTdb), a comprehensive scLT database with multifunctional modules for the analysis of gene expression, clonal compositions, fate outcomes, lineage relationships and potential regulators of cell fate determination. We collected 109 datasets including three species, 13 tissue sources, 2.8 million cells and 36 scLT technologies. There are three special features of scLTdb. First, scLTdb is a lineage tracing specific database, providing an atlas of fate-resolved cells that are overlooked by traditional single-cell RNA sequencing (scRNA-seq) datasets. Second, scLTdb provides interactive modules for cell fate data re-analysis, serving as a high-confidence resource for fate mapping and lineage reconstruction. Third, scLTdb allows users to identify fate-related gene expression or chromatin accessibility, revealing the molecular difference between fate biases. In addition, users can upload their own data and perform clonal fate analysis. Thus, scLTdb provides a powerful tool for the analysis of existing and unpublished scLT datasets, which will facilitate the understanding of cellular behavior and the mechanisms underlying cell fate determination in health and diseases.

## Materials and methods

### Data collection

To assemble a comprehensive repository, we manually curated scLT datasets from literature up to June 2024. We downloaded scLT datasets from several databases, including the Gene Expression Omnibus, NCBI BioProject and Zenodo (21,22). Each dataset includes transcriptome or epigenomics information paired with cell lineage barcodes. We also manually reviewed the literature and supplemental materials to curate the meta-information of each dataset, including information on species, barcode types, scLT technologies and tissue sources (Supplementary Table S1).

### Data pre-processing

We employed a four-step process to pre-process the scLT data. (i) We removed low-quality cells based on the criteria in the original study. (ii) We normalized the data using the ‘NormalizeData’ function from the R package Seurat (version 4.4.0) (23). (iii) We reduced data dimensions and visualized cells using principal component analysis and Uniform Manifold Approximation and Projection (UMAP) with the ‘RunPCA’ and ‘RunUMAP’ functions from the R package Seurat (24). (iv) For cell type annotation, we annotated cells based on the expression of known cell markers from the CellMarker2.0 database (25) or cell identity information provided by the original studies.

### Pseudo-time inference

We utilized the partition-based graphabstraction (PAGA) method, implemented in the omicverse Python package (version 1.5.9), to construct trajectories and infer pseudo-time for each dataset (26,27). To this end, we employed the ‘ov.single.TrajInfer’ function to construct a diffusion map, with the parameter ‘n_comps’ set to 50, and then applied the ‘ov.Traj.inference’ function to calculate pseudo-time values.

### Identification of high-confidence barcodes (clones)

Due to technique issues, some barcodes label more than one cell at the initial barcoding stage. These barcodes cannot represent cells derived from a single progenitor cell, termed clone. Therefore, it is required to filter these barcodes before clonal analysis. We apply the method from the original paper to present high-confidence barcodes in scLTdb.

### Alignment of single-cell lineage barcodes

The scLT experiments generate a FASTQ file containing lineage barcodes and their corresponding cell indexes in single-cell RNA (or single-cell ATAC) data. Theoretically, one cell only has one lineage barcode, so one cell index should match only one barcode sequence. However, due to sequencing errors, a cell index might match multiple barcodes. Therefore, it is necessary to align unique barcodes with each cell index. In our database, we employed the methods from the original study to identify unique barcodes per cell index.

### Clone analysis and visualization

To analyze and visualize lineage barcodes within scLT data, we wrapped all analysis steps into an R package FateMapper (https://github.com/jiang-junyao/FateMapper). FateMapper primarily incorporates four functionalities: (i) The ‘cal_clone_size’ function is used to calculate clone size in each scLT dataset. Clone size represents the number of cells carrying the same clonal barcode. The clone size information is visualized through R package ggplot2 (version 3.3.6). (ii) The ‘fate_mapping’ function is used to map cell fate bias of targeted population by visualizing barcode propagation across various cell types. This function calculates a specific barcode’s ratio of the targeted population to all cell types. The equation for calculating barcode’s ratio is as below:


\begin{eqnarray*} &&{normalized\ value\ of\ barcode\ \beta \ in\ cell\ type\ A} \nonumber\\ &&\quad= \frac{{counts\ of\ barcode\ \beta \ in\ cell\ type\ A}}{{counts\ of\ barcode\ \beta \ in\ all\ cell\ types}}.\end{eqnarray*}

(iii) The ‘lineage_relationship’ function is used to analyze and visualize the lineage relationships between various cell types. It calculates the Spearman correlation of barcode signatures between each cell type pair, and then visualizes the results via the ‘pheatmap’ function from R package pheatmap (version 1.0.12) (28). (iv) The ‘plot_clone_embedding’ function is used to visualize a single clone or a set of clones with the same fate bias on the embedding plot using the R package ggplot2 (29).

### Clone fate bias analysis

Clone fate bias represents the differentiation preference of progenitors into specific downstream cell types. For studies that defined clone fate bias, we directly used this information to label clone fate bias in our database, ensuring that our analysis is consistent with the original study. For other studies, we utilized the ‘clone_fate_bias’ function from the R package FateMapper to evaluate the fate bias of each clone. This function uses Fisher’s exact test to quantify the statistical significance of a clone’s occupancy within a set of cell types, compared with what would be expected from a random sampling of cells. We subsequently employed false discovery rate (FDR) adjustment, based on the Benjamini–Hochberg procedure, to the *P*-values obtained from the ‘clone_fate_bias’ analysis. Through this adjustment, we identified clones exhibiting significant fate bias (FDR < 0.05).

### Identification and visualization of fate-related DEGs or DARs

To identify differentially expressed genes (DEGs) for cells with different fate biases in scRNA-seq data, we utilized the ‘FindMarker’ function from the R package Seurat. The ‘test.use’ parameter of ‘FindMarker’ was set to ‘negbinom’ (24). To identify differentially accessible regions (DARs) between different fate biases in single-cell ATAC sequencing (scATAC-seq) data, we adjusted ‘test.use’ parameter of ‘FindMarker’ to ‘wilcox’. Genes and peaks with *P*-value <0.05 and absolute value of Log_2_FoldChange >0.5 were selected as DEGs and DARs. Peak related gene was annotated by ‘get_related_genes’ function from R package IReNA (version 1.0.0) (30). ‘Doheatmap’ function from R package Seurat and ‘ggplot’ function from R package ggplot2 were used to visualize the DEGs and DARs. We used R package ClusterProfiler (version 3.18.1) to perform functional enrichment analysis that is based on gene ontology (GO) database. Parameter ‘pvalueCutoff’ was set to 0.05 (31,32).

### Motif enrichment analysis

The ‘matchMotifs’ function from the R package ‘motifmatchr’ (version 1.16.0) was utilized to scan for enriched motifs on each DARs and to calculate motif enrichment scores (33). The parameter ‘p.cutoff’ was set to 5e−05. The position weighted matrix was obtained from R package chromVARmotifs (version 0.2.0, https://github.com/GreenleafLab/chromVARmotifs).

### Implementation

The scLTdb has been developed as an interactive web application utilizing the Shiny framework of R (https://shiny.rstudio.com/). In addition, the aesthetics of our web interface was enhanced using several R packages, including ggplot2 for sophisticated static graphics, shinyWidgets for interactive elements, plotly for dynamic visualizations and shinyjqui for user interface components (34). The scLTdb is freely available at https://scltdb.com.

## Results

### Scheme of scLTdb

**Figure 2.**
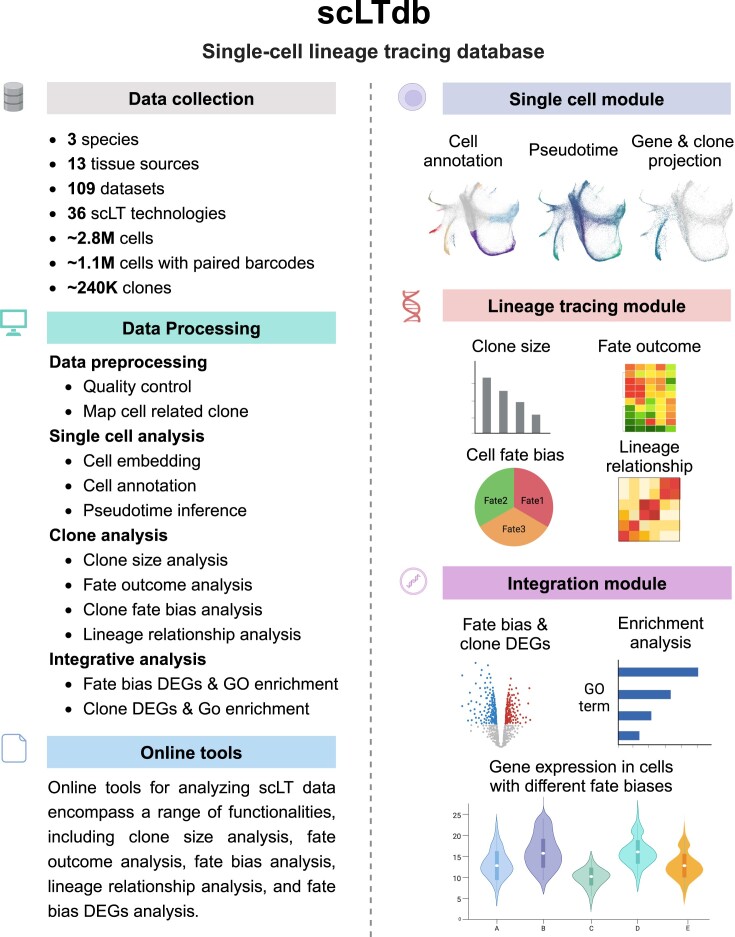
Schematics of scLTdb.

scLTdb provides a comprehensive overview of 109 datasets published before June 2024. We analyzed each scLT dataset using a standard pipeline and developed three functional modules to facilitate browsing and analysis of each dataset (Figure 2). The single-cell module offers various features for transcriptomic or epigenomic analysis, such as cell type annotation, pseudo-time inference, cell clone embedding and gene expression projection. The lineage tracing module provides interactive tools for users to analyze clone sizes, cell fate outcomes, clone fate biases and lineage relationships. The integration module presents the integrated analysis of scRNA-seq and lineage tracing data, including DEG analysis between cells with different fate outcomes. Users can visualize the expression of gene of interest across different cell fate biases within this module. Additionally, scLTdb provides online tools for users to analyze their own scLT data, and a step-by-step tutorial is also available for users to explore scLTdb (Figure 2).

### Summary of scLTdb

**Figure 3.**
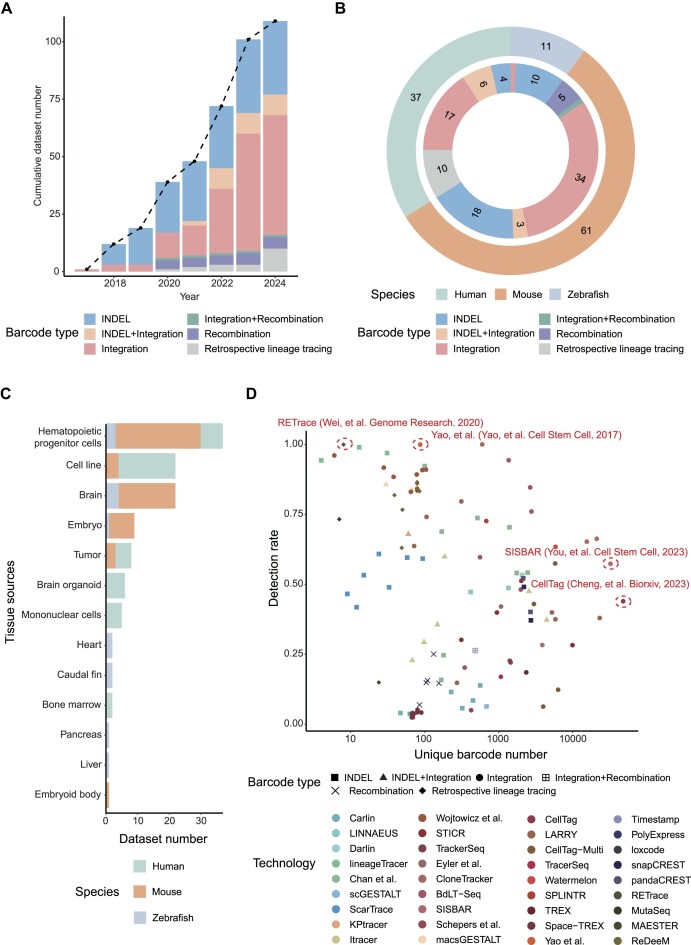
Data summary of scLTdb. (**A**) Bar plot shows the cumulative scLT dataset number from 2017 to 2024, colors represent barcode types. (**B**) Number of datasets summarized by species and barcode types. (**C**) Bar plot illustrates number of scLT datasets across different tissue sources. (**D**) Dot plot shows the barcode detection rate and unique barcode number of each dataset. Colors represent scLT technologies, shapes represent barcode types.

The scLTdb provides an extensive compilation of scLT studies conducted from 2017 to June 2024 (Supplementary Table S1). Currently, the database encompasses 109 datasets that employ different barcoding types, and with a dramatic growth over the years (Figure 3A). The highest increase of 29 datasets occurred in 2023, which highlights the growing impact of scLT in various fields, such as immunology and developmental biology (Figure 3A). Among these, the integration barcode type is the most frequently used method, resulting in the generation of 52 scLT datasets. In addition, mouse and human are the two most studied species, consisting of 55.96% and 33.94% of all experiments, respectively (Figure 3B).

The scLTdb comprises 13 distinct tissue sources. Hematopoietic progenitor cells possess the largest number of datasets, with a total of 37 datasets (mouse 27, human 7 and zebrafish 3), cell line (human 18 and mouse 4) and brain (mouse 18 and zebrafish 4) come in the second place with 22 (Figure 3C).

The resolution and robustness of scLT experiments largely depend on the number of distinct barcodes and the ratio of cells carrying detectable barcodes. To compare these two key parameters between scLT technologies, we calculated barcode diversity (unique barcode number) and barcode detection rate (the proportion of cells with barcodes that can be detected using scRNA-seq) for each dataset. The top two datasets that have the highest number of unique barcodes are generated by CellTag (48 234 unique barcodes) and SISBAR (32 549 unique barcodes) (35,36), indicating the high resolution of the integration barcode type used in these two studies (Figure 3D). Although datasets generated by the retrospective lineage tracing-based method (RETrace) and the integration-based method (38) have 100% detection rate, these technologies have limited number of unique barcodes (only 8 and 87 barcodes, respectively) (Figure 3D) (37,38). Summary for scLT technologies and dataset details, including advantages, limitations, quality control and data processing procedures, can be found in the supplementary material (Supplementary Tables S1–S3).

### Searching and querying datasets in scLTdb

**Figure 4.**
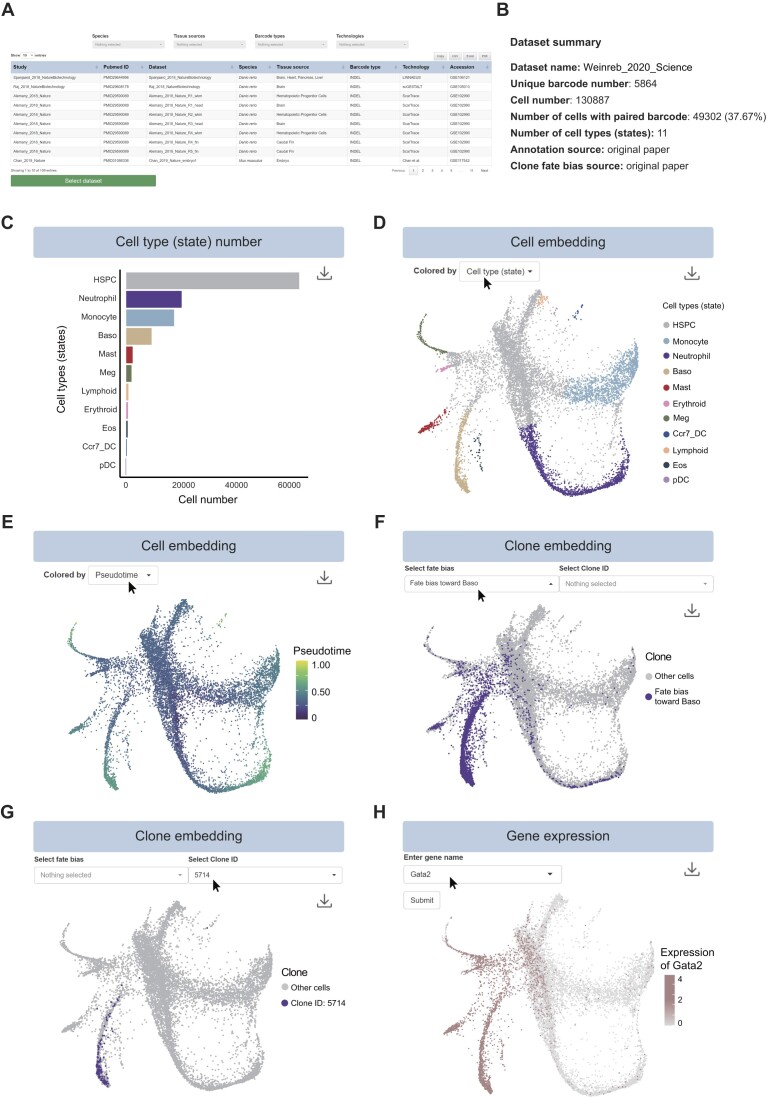
Web interface of search page and single-cell module. (**A**) Data search page of scLTdb. (**B**) Data summary table of selected dataset. (**C**) Bar plot of cell number in each cell type (state). (D and E) Single-cell embedding plot, colored by cell types (states) or pseudo-time. The mouse pointer highlights an interactive button that can be used to change the color representations for cell types (states), pseudo-time, sample information and group information. (**F**) Single-cell embedding plot colored by clones with fate bias toward basophil (Baso). The mouse pointer highlights an interactive button, which facilitates the plotting of a set of clones with same fate bias on the embedding plot. (**G**) Single-cell embedding plot, colored by a specific single clone (clone ID: 5714). The mouse pointer highlights an interactive button, which facilitates the plotting of a single clone on the embedding plot. (**H**) Single-cell embedding plot, colored by Gata2 expression. The mouse pointer highlights an input box for entering gene name.

In the ‘Search’ interface of scLTdb, users can readily query scLT datasets based on options such as ‘Species’, ‘Tissue source’, ‘Technology’ and ‘Barcode type’. The results are presented in an interactive table including study name and PubMed ID of the datasets-related paper. Users can select a dataset in the table and click the ‘Select dataset’ button to access the analysis results of the selected dataset (Figure 4A).

### Functional modules

The scLTdb offers three functional modules that enable users to re-analyze and visualize scLT datasets through the web interface, including single-cell module, lineage tracing module and integration module. We selected a scLT dataset of mouse hematopoiesis to demonstrate the usability of these interactive modules. This dataset was generated by an integration barcode based scLT technology (LARRY), to track the differentiation of hematopoietic stem and progenitor cells (HSPCs) *in vitro* (39). After barcoding and cell division, cells were sampled immediately or after differentiation, which aims to integrate the initial states of cells with their fate outcomes on transcriptional landscape (39). To reduce the time for data loading, if the selected dataset exceeds 10 000 cells, the embedding plot in the single-cell module and the violin plot in the integration module will only show a sampled dataset of 10 000 cells.

### Single-cell module

Transcriptomic or epigenomic analysis can generate a global picture of cell identities and cell state dynamics within scLT datasets. Therefore, scLTdb provides single-cell module, which offers a variety of functions for visualizing and re-analyzing gene expression, DNA methylation and chromatin accessibility in selected datasets.

In this module, users can first have an overview of the selected dataset by referring to a data summary table (Figure 4B). This table contains key information of the selected dataset, including dataset name, unique barcode number, cell number, number of cells with paired barcodes, barcode detection rate, number of cell types (states), annotation source and clone fate bias source (Figure 4B). In addition, this module also offers a bar plot to display cell count of each cell type (Figure 4C). Additionally, users can visualize a single-cell embedding plot colored by cell types (states) (Figure 4D). This plot allows users to gain basic information about cell identities and compositions. By clicking on the ‘colored by’ button, users can choose to replace the color coding in the embedding plot with sample information, time information or pseudo-time values inferred using the PAGA algorithm (Figure 4E, and Supplementary Figure S1A and B) (26). This pseudo-time analysis enables users to visualize the putative developmental trajectory of cells. Then, users can project cell lineage barcodes on the single-cell embedding plot to visualize the developmental trajectory of all clones derived from progenitors with the same fate bias. For example, when selecting the ‘Baso biased’ clones, scLTdb generates an embedding plot that highlights cells (purple dots) derived from HSPC with fate bias into basophil lineage (Figure 4F). When a single clone with the ‘Baso biased’ fate is chosen, scLTdb produces an embedding plot that is colored solely based on that specific clone (Figure 4G). In addition to cell clone projection, this module also provides an interactive function that allows users to visualize gene expression on the embedding plot by inputting the gene name (Figure 4H). Furthermore, we also provide marker gene information, enabling users to visualize the top five DEGs in each cell type (Supplementary Figure S1C).

### Lineage tracing module

Lineage tracing experiments provide the ground truth information of cell fate and cell lineage. Therefore, scLTdb provides a lineage tracing module that allows users to visualize and re-analyze cell fate outcomes and lineage relationships within scLT datasets.

**Figure 5.**
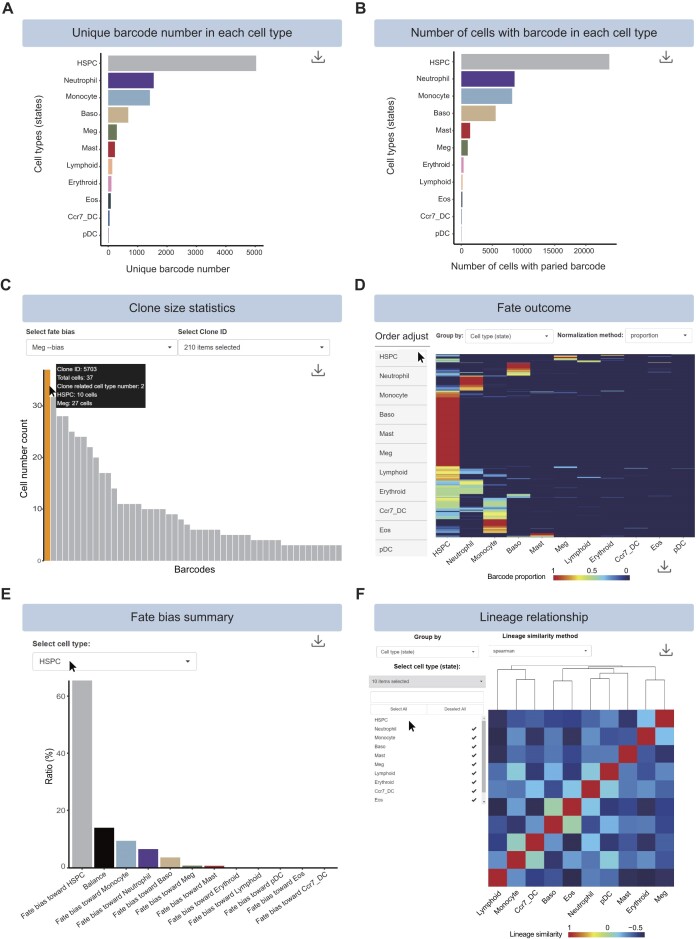
Web interface of lineage tracing module. (**A**) Bar plot for barcode numbers in each cell type (state). (**B**) Bar plot for numbers of cells with detected barcodes in each cell type (state). (**C**) Bar plot of clone size. Users can put mouse pointer on the bar to check clone ID, clone size and the number of cell types in selected clones. (**D**) Heatmap of fate outcomes. Each column represents a cell type (state), each row represents a barcode and value represents barcode enrichment toward cell types (states). Users can manipulate the order of cell type columns in the fate outcome heatmap by dragging the cell type boxes up or down on the left panel. (**E**) Fate bias summary in HSPC. Bar plot displays the fractions of HSPC fate biases toward other cell types. (**F**) Heatmap of lineage relationships, values represent Spearman correlation among cell types (states). Users can select cell types of interest to infer lineage relationships by using the button to the left panel.

The ‘Barcode statistics’ function provides a quantitative overview of cell clone information. User can generate two bar plots that illustrate the number of unique barcodes and the number of cells with paired barcodes in each cell type (Figure 5A and B). This function can also visualize the number of cells carrying the same clonal barcode (i.e. clone size), which can infer the events of clonal expansion or cell–cell competition. Additionally, it generates a bar plot of clone sizes in a descending order, from the largest to the smallest. Clone size, clone ID and number of cell types in the selected clone are displayed upon mouse hover over each bar (Figure 5C). Moreover, the lineage tracing module provides the ‘Clone size ranges’ function, which enables users to visualize clone size across different ranges (Supplementary Figure S2A). Finally, users can explore the correlation between clone size and the number of clone-related cell types through the violin plot in the lineage tracing module (Supplementary Figure S2B).

The ‘Fate outcome’ function aims to map cell fate of targeted populations by visualizing the barcode propagation between progenitors and their daughter cells. Users can browse cell fate outcomes across different cell types or group information (such as time points) and manipulate the order of cell type columns in the fate outcome heatmap (Figure 5D, and Supplementary Figure S2C). This function can calculate the differentiation bias of progenitors toward downstream specific cell types, and thus, generate a quantitative analysis of cell fate bias. As an example, we display the fate biases of HSPC in mouse hematopoiesis (39). The bar plot indicates that the majority of HSPCs (65.46%) display an ‘HSPC-biased’ fate, indicating that these cells prefer to self-renew or keep inactive rather than differentiate (Figure 5E). Additionally, 9.3% HSPCs exhibit a ‘Monocyte-bias’ fate, indicating their differentiation preference into monocytes (Figure 5E).

The ‘Lineage relationship’ function is used to compare the lineage similarity between cell types (states) based on the number and frequency of barcodes present in each cell type (state) or group (such as in different time points) (Figure 5F, and Supplementary Figure S2D). If two cell types share many barcodes at similar frequencies, they are likely to have arisen from a common developmental pathway; if not, they probably developed more independently. Users can calculate the lineage similarity of barcodes between cell types and generate a heatmap to visualize lineage relationships (Figure 5F). Lineage tree is displayed at the top of the heatmap, illustrating the relationship and developmental hierarchy. For example, eosinophils and basophils are located in the same branch, indicating a close lineage relationship between these two cell types.

Furthermore, scLTdb offers phylogenetic trees for Cas9-based and retrospective lineage tracing datasets that are suitable for phylogenetic analysis (Supplementary Figure S2E). Users can download the raw lineage barcode sequences from the above datasets on the download page and use them to construct phylogenetic trees.

### Integration module

Because lineage tracing data can provide the ground truth cell lineage information, integrative analysis of lineage tracing and gene expression or chromatin accessibility can be used to identify potential molecular markers or regulators for cells with specific fate biases. Additionally, when comparing single clones of different sizes, one may potentially uncover the molecular programs driving cell proliferation, cell fitness or differentiation. Therefore, scLTdb provides integration module that offers a range of functions to perform integrative analysis of cell fate-related genes or chromatin accessible regions.

**Figure 6.**
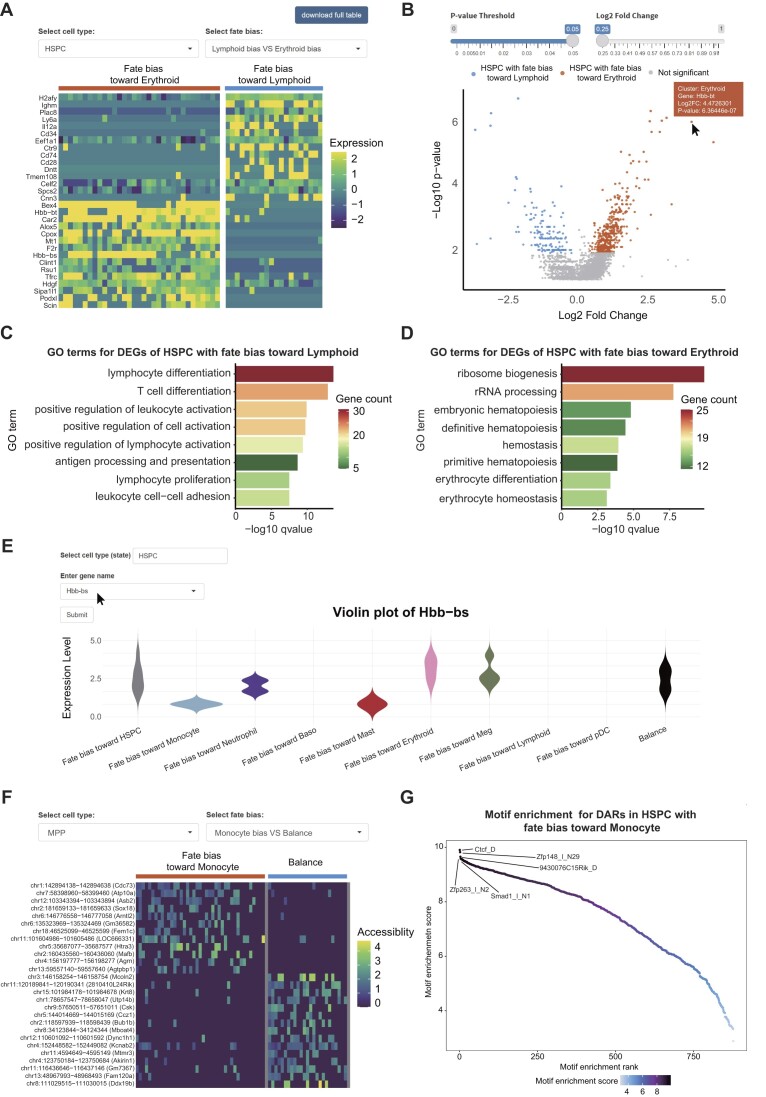
Web interface of integration module. (A and B) Heatmap and volcano plot of DEGs between HSPCs with fate bias toward lymphoid lineage and HSPCs with fate bias toward erythroid lineage. The mouse pointer highlights the presence of two switches that can be employed to adjust the threshold of DEGs. (**C**) Bar plot of GO enrichment analysis for DEGs of HSPCs with fate bias toward lymphoid lineage. (**D**) Bar plot of GO enrichment analysis for DEGs of HSPCs with fate bias toward erythroid lineage. (**E**) Violin plot of Hbb-bs expression for HSPCs with different fate biases. The mouse pointer highlights an input box for entering gene name. (F–G) Heatmap of DARs and motif enrichment analysis for multipotent progenitors (MPP) with balanced fate outcomes and fate bias toward monocyte lineage.

This module can calculate DEGs for different fate biases. Users can identify the DEGs associated with fate biases in two steps: (i) select a cell type using the ‘Choose cell type’ button and (ii) choose a cell fate bias group using the ‘Choose fate bias’ button (Figure 6A). We provide heatmap and volcano plot to present the results of DEGs analysis. For volcano plot, users can define the significant DEGs by adjusting the threshold of *P*-value and fold change (Figure 6B). For instance, we identified DEGs between HSPCs with fate bias toward lymphoid lineage and HSPCs with fate bias toward erythroid lineage. The heatmap displays the top 15 DEGs in each group, including Hbb-bt and Hbb-bs, which are markers of erythroid lineage (24). Conversely, Cd28, a gene that is required for T cell activation, was found to be upregulated in lymphoid-biased HSPCs (Figure 6A) (40,41). Moreover, scLTdb provides GO enrichment analysis for identified DEGs. The results indicate that lymphoid-biased HSPCs are enriched for genes in lymphocyte differentiation, T cell differentiation and positive regulation of lymphocyte activation, thereby suggesting their differentiation potential toward the lymphoid lineage (Figure 6C). On the other hand, erythroid-biased HSPCs show gene enrichment for erythrocyte differentiation, indicating their differentiation potential toward the erythroid lineage (Figure 6D). Finally, this module also offers an interactive violin plot to visualize the expression of specific genes across different fate biases within a given cell type. Users can specify the cell type and gene of interest through the input box above the violin plot (Figure 6E). For example, we chose HSPC as the target cell type and selected the erythroid marker Hbb-bs. The results indicate a high expression of Hbb-bs in HSPCs with fate bias toward erythroid lineage.

For datasets with cell fate information and paired scATAC-seq (assay for transposase-accessible chromatin using sequencing) data (11), this module can also calculate DARs for cells with different fate biases. We provide heatmap to present fate-related open chromatin regions that are enriched in specific fate bias, and then performed motif enrichment analysis to show the potential DNA-binding proteins of DARs (Figure 6F and G). Taken together, the integration module allows users to not only identify genes or genomic regions that are potentially involved in cell fate decisions but also predict the functional differences between cells with different fate biases.

### Data download and online tools

**Figure 7.**
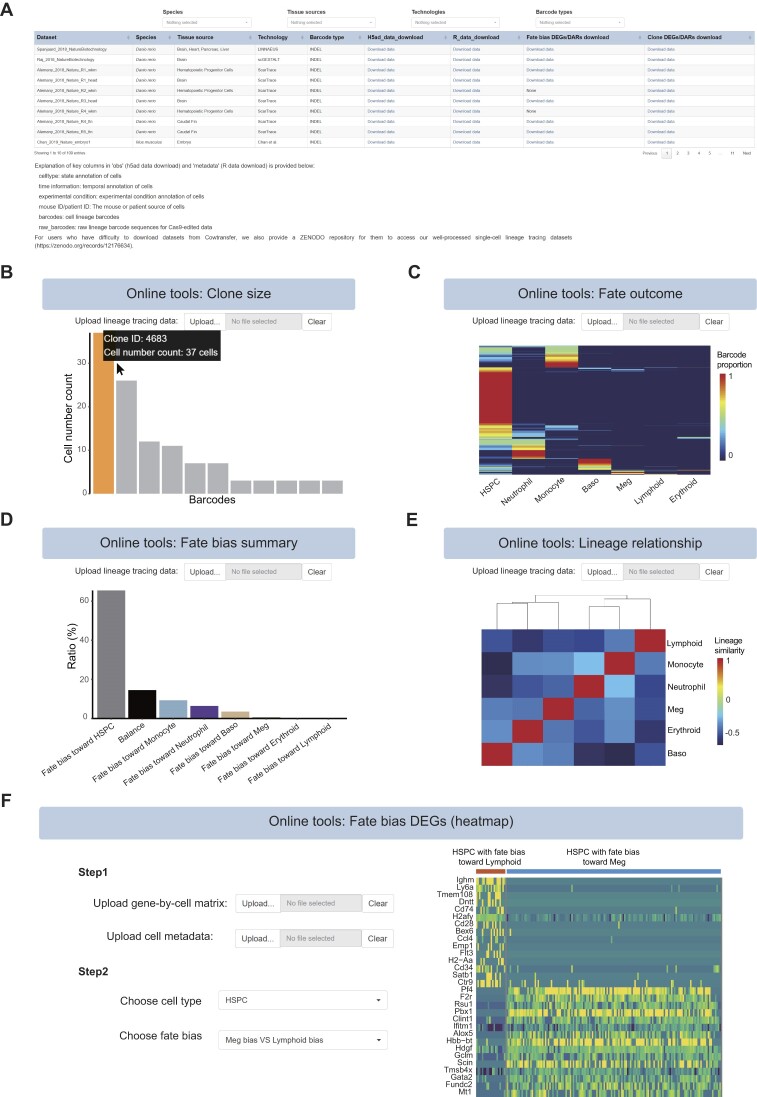
Web interface of download page and online tools. (**A**) Web interface of download page. Users can click hyperlinks (‘Download data’) to access the download pages of datasets. (**B**) Clone size analysis function within the online tools of scLTdb. Users can put mouse pointer on the bar to check clone ID and clone size. (**C**) Fate outcome function within the online tools. Each column represents a cell type (state), and each row represents a barcode. (**D**) Fate bias summary function within the online tools. Bar plot displays the fractions of HSPC fate biases toward other cell types. (**E**) Lineage relationship within the online tools. Value represents Spearman correlation among cell types (states). (**F**) Heatmap of DEGs between different cell fate biases. Users need to first upload the gene-by-cell matrix (peak-by-cell matrix) and cell metadata, then select cell type and fate bias to initiate the analysis.

The scLTdb facilitates the download of well-processed scLT datasets. In the ‘Download’ section, scLTdb offers an interactive table for users to search datasets using various criteria, such as ‘Species’, ‘Tissue source’, ‘Barcode type’ and ‘Technology’. After performing queries, users can obtain the download link for the selected dataset in H5ad format from the ‘H5ad data download’ column, or in Seurat object format from the ‘R data download’ column (Figure 7A). Additionally, download links for the table of fate bias DEGs or DARs are also provided in the ‘Fate bias DEGs/DARs download’ column (Figure 7A). This table contains the full analysis results of fate bias DEGs or DARs, including *P*-values and fold changes of DEGs.

For users who need to process their own scLT data, we have developed online tools on our web server for clone analysis. On the ‘Online tools’ page, users can upload a clone matrix where rows represent clones and columns represent cell types by clicking on the ‘Upload’ button. Then, scLTdb will generate clone analysis results including information on clonal size statistics, cell fate outcomes, fate bias summary, lineage relationships and fate-related genes (Figure 7B–F).

## Discussion

The gold standard for inferring lineage relationship is lineage tracing. As an emerging technique, scLT allows for simultaneous detection of cell states (transcriptome or epigenomics) and DNA barcodes that are used for cell labeling and lineage inferring, which provides a powerful platform to discover new cell lineages and cell fate regulators.

To collect and analyze growing scLT data, we built the first scLTdb, which can provide the ground truth cell lineage information (via barcodes). The scLTdb involves 13 tissue sources, including multiple biological processes such as embryogenesis, hematopoiesis, neurogenesis and tumorigenesis. This broad coverage will benefit a large number of users in these fields by allowing them to explore cells of interest that are annotated with cell fate and gene expression, which will help users to generate new ideas and hypotheses. Computational methods could predict cell fate changes after experimental or *in silico* perturbations. The scLTdb with the ground truth cell lineage information will be an important resource for users to benchmark existing methods and develop new computational models for cell fate prediction and lineage inference.

In the future, we plan to extend scLTdb as follows. We will continue to collect publicly available scLT data to expand the species, tissues and diseases covered in scLTdb. In addition, we and our collaborators are performing scLT experiments and will continuously update the data on our webpage in the coming years. Because scLT is a fast-growing field, scLTdb will integrate data of next-generation technologies, such as single-cell multiomics lineage tracing and spatial lineage tracing, and launch more analysis modules. In conclusion, scLTdb is a unique resource for users to investigate cell fate determination, clonal behavior and developmental pathways in development and disease.

## Supplementary Material

gkae913_Supplemental_Files

## Data Availability

The scLTdb is freely available at https://scltdb.com.
