# Supplementary material for: scLTdb: a comprehensive single-cell lineage tracing database
Source: Nucleic Acids Res. 2024 Oct 29;53(D1):D1173–85. doi: 10.1093/nar/gkae913 (PMC11701529; doi:10.1093/nar/gkae913)
Supplement: gkae913_Supplemental_Files [file gkae913_supplemental_files.zip › Supplementary figures and legends.pdf]

# Supplementary Figure1

A

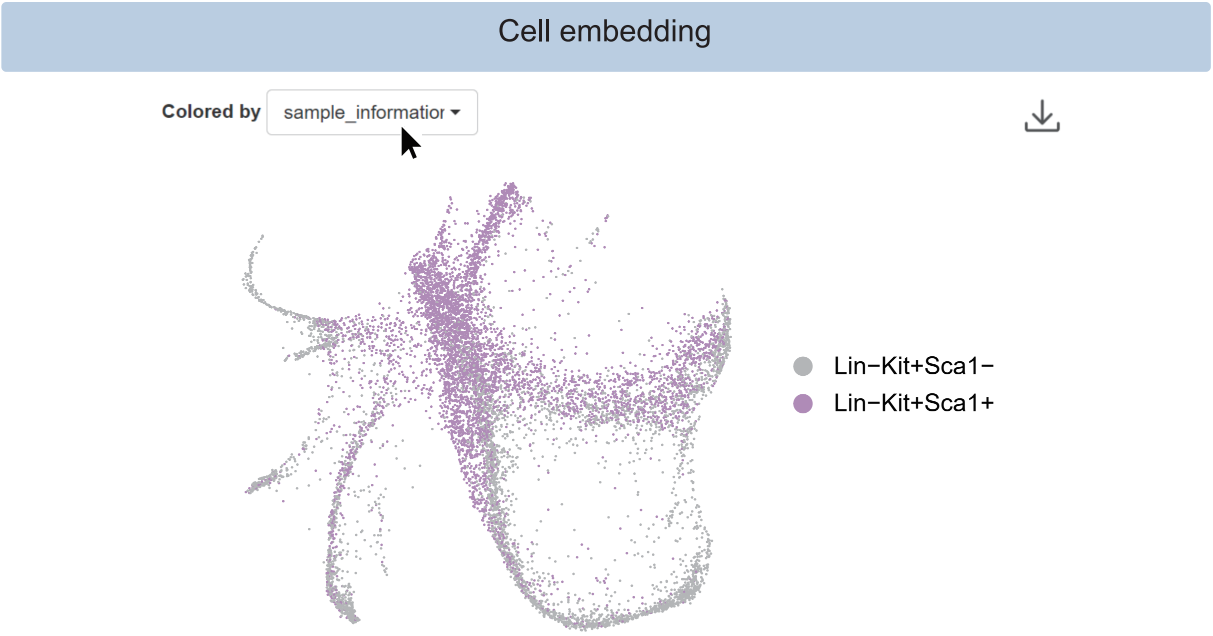


B

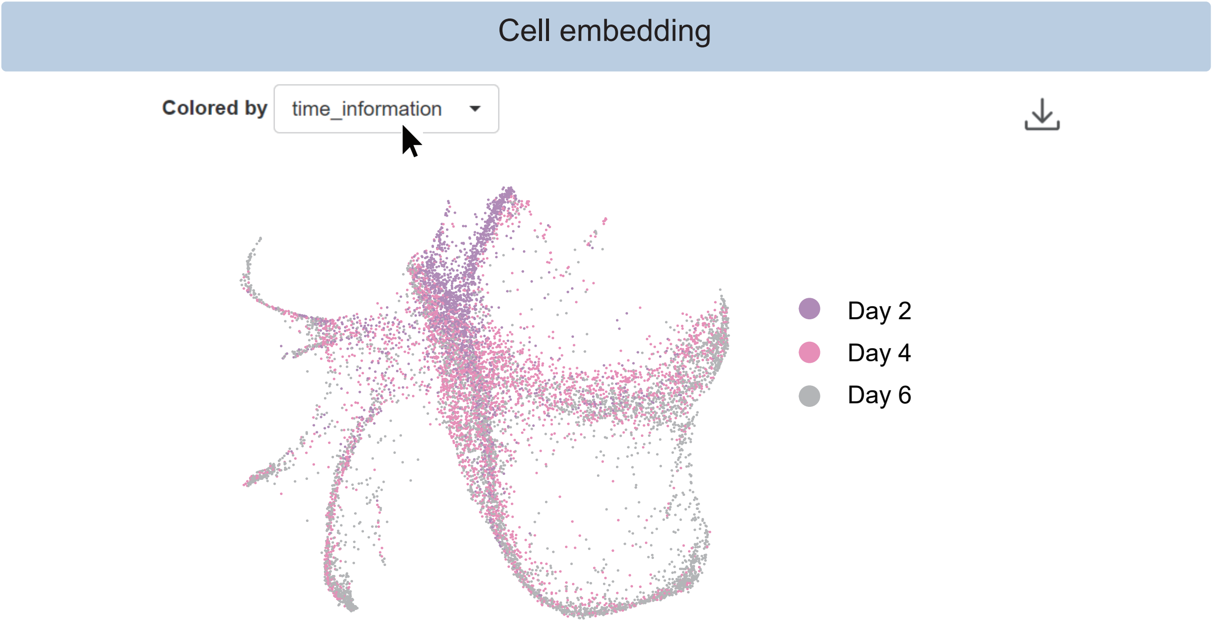


C

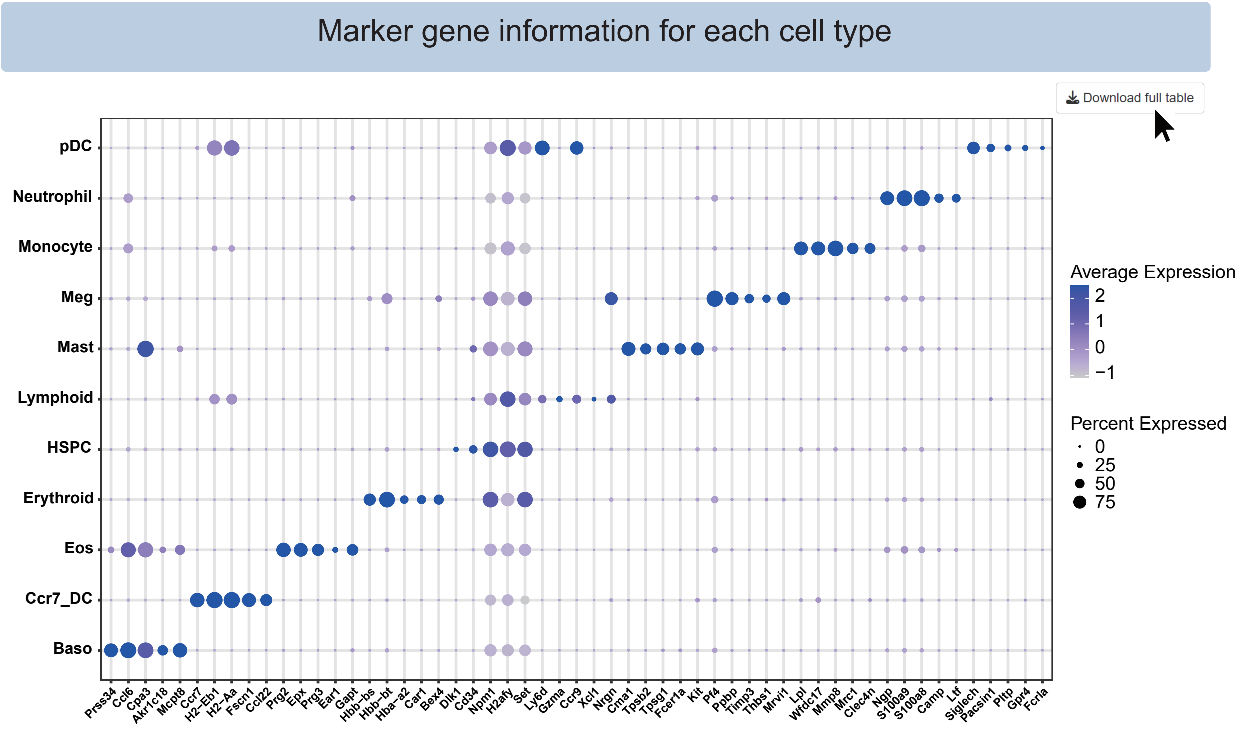

**Supplementary Figure 1. Web interface of single cell module.** Single cell embedding plot, colored by sample information (A) and group information (time points) (B). The mouse pointer highlights an interactive button that can be used to change the color representations. (C) 'Marker gene information for each cell type' function. The mouse pointer highlights the button to download the full table of marker genes.

# Supplementary Figure2

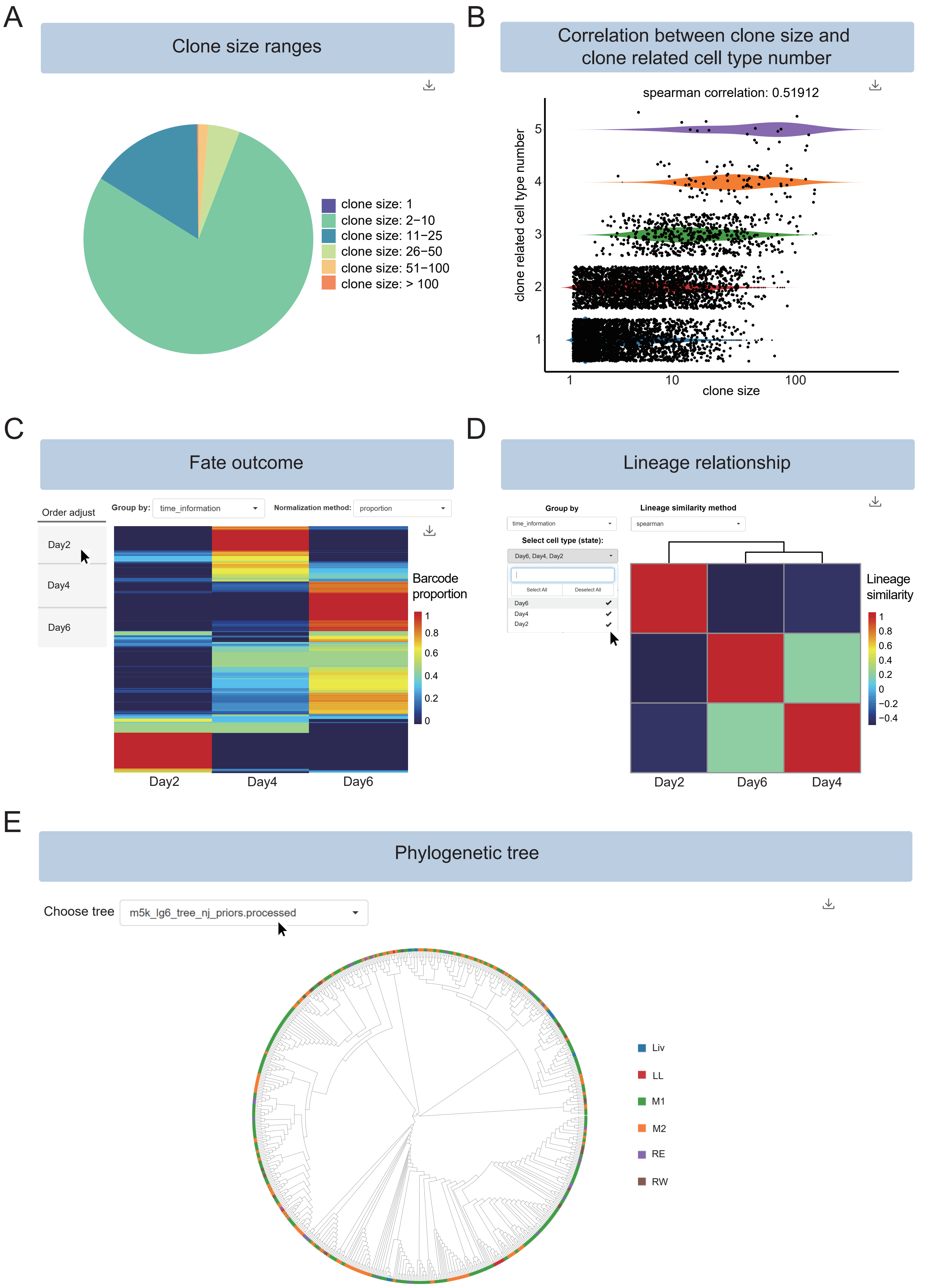

**Supplementary Figure 2. Web interface of lineage tracing module.** (A) Pie charts of clone size range statistics. (B) Violin plots of clone size (cell number) and clone-related cell type number. (C) Heatmap of fate outcomes. Each column represents one time point, and each row represents a lineage barcode. Value represents barcode enrichment toward time points. Users can manipulate the order of columns in the fate outcome heatmap by dragging the boxes on the left panel. (D) Heatmap of lineage relationships. Value represents spearman correlation among time points. (E) Phylogenetic tree for the "Quinn_2021_Science_5k" dataset. Each tree node represents a single cell, and each color represents a sample.
